# Photonic heat transport in three terminal superconducting circuit

**DOI:** 10.1038/s41467-022-29078-x

**Published:** 2022-03-23

**Authors:** Azat Gubaydullin, George Thomas, Dmitry S. Golubev, Dmitrii Lvov, Joonas T. Peltonen, Jukka P. Pekola

**Affiliations:** QTF Centre of Excellence, Department of Applied Physics, Aalto University School of Science, P.O. Box 13500, FI-00076 Aalto, Finland

**Keywords:** Thermodynamics, Qubits

## Abstract

We report an experimental realization of a three-terminal photonic heat transport device based on a superconducting quantum circuit. The central element of the device is a flux qubit made of a superconducting loop containing three Josephson junctions, which can be tuned by magnetic flux. It is connected to three resonators terminated by resistors. By heating one of the resistors and monitoring the temperatures of the other two, we determine photonic heat currents in the system and demonstrate their tunability by magnetic field at the level of 1 aW. We determine system parameters by performing microwave transmission measurements on a separate nominally identical sample and, in this way, demonstrate clear correlation between the level splitting of the qubit and the heat currents flowing through it. Our experiment is an important step towards realization of heat transistors, heat amplifiers, masers pumped by heat and other quantum heat transport devices.

## Abstract (web summary)

Quantum heat transport devices are currently intensively studied. Here, the authors report the photonic heat transport modulated by superconducting qubit in a three-terminal device. Flux dependent heat power correlates with microwave measurements.

## Introduction

Recent achievements in superconducting circuit QED techniques in combination with ultrasensitive nanoscale thermometry^1–4^ stimulated theoretical discussion and experimental realization of on-chip refrigerators^5–8^, quantum heat engines^9^, heat rectifiers^10^, interferometers^11^, and other thermal devices. Superconducting loops containing Josephson junctions and coupled to superconducting resonators are essential parts of such systems because they allow one to control photonic heat currents by magnetic field. Recent examples based on this architecture include quantum heat valve based on the resonator-qubit-resonator assembly^12^ and heat rectifier with unequal frequency resonators^13^.

**Fig. 1:**
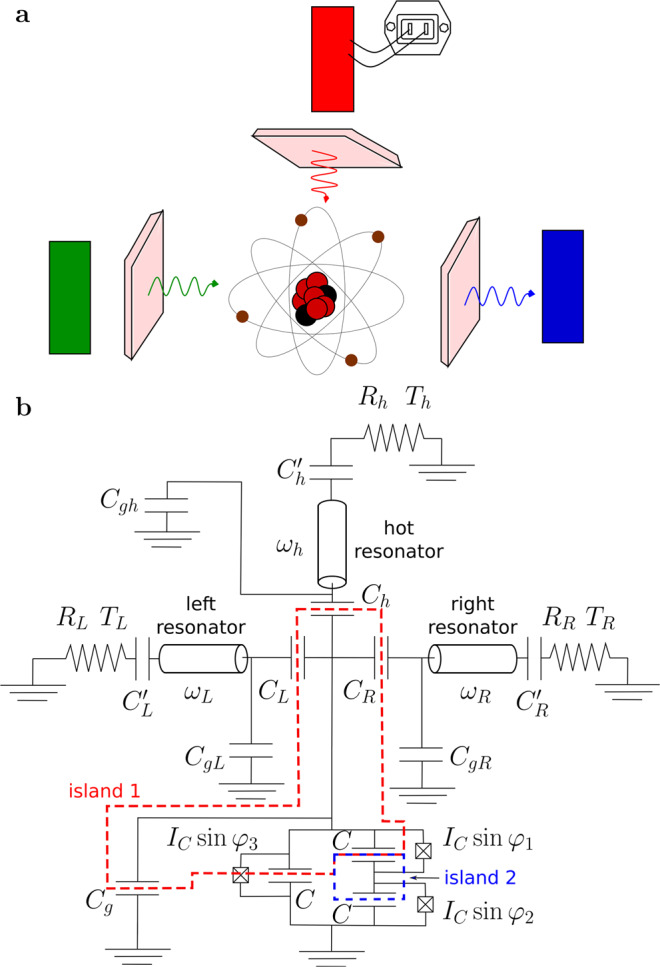
Three terminal device with circuit schematic used for simulations. **a** Heat transport in a three terminal system containing an artificial atom. **b** Schematics of a superconducting circuit, which models our device and realizes the heat transport experiment sketched in (**a**).

So far most experiments in this field have been carried out in two terminal devices. However, according to the theory, moving to three terminal setup^14^ should open up new opportunities. Indeed, there exist theory proposals for thermal transistors^15–18^, heat amplifiers^19,20^, quantum absorption refrigerators^21,22^ and thermally pumped masers^23,24^, which are heat engines from thermodynamical point of view, in this configuration. Motivated by these ideas, we have fabricated a three terminal heat transport device containing an Xmon qubit, which acts as a tunable element and controls photonic heat currents between the terminals. Basic schematics of our device is shown in Fig. 1. Its main element is the flux qubit made of a superconducting loop containing three identical Josephson junctions. Qubit frequency can be tuned by application of magnetic field, which induces the magnetic flux inside the loop. The qubit is coupled to three resonators—the left, the right and the hot one, which filter thermal noises emitted by three ohmic resistors. The temperature of the hot resistor can be varied by application of the heating current to it and the temperatures of the two other resistors are monitored by electronic thermometers. Heating the hot resistor we bring the whole system into the non-equilibrium steady state and vary the heat currents by magnetic flux. In this way, we demonstrate the control of the photonic heat power at the level of 10^−18^ W. In order to get more information about the system parameters, we have performed microwave transmission measurements on the nominally identical twin sample. We have also developed a theory model, which reasonably well explains the experimental findings. We are confident that further technological developments will soon permit practical implementation of the interesting theoretical proposals mentioned above and investigation of the effects of quantum coherence on the performance of heat transport devices^22^.

## Results

### Experimental

**Fig. 2:**
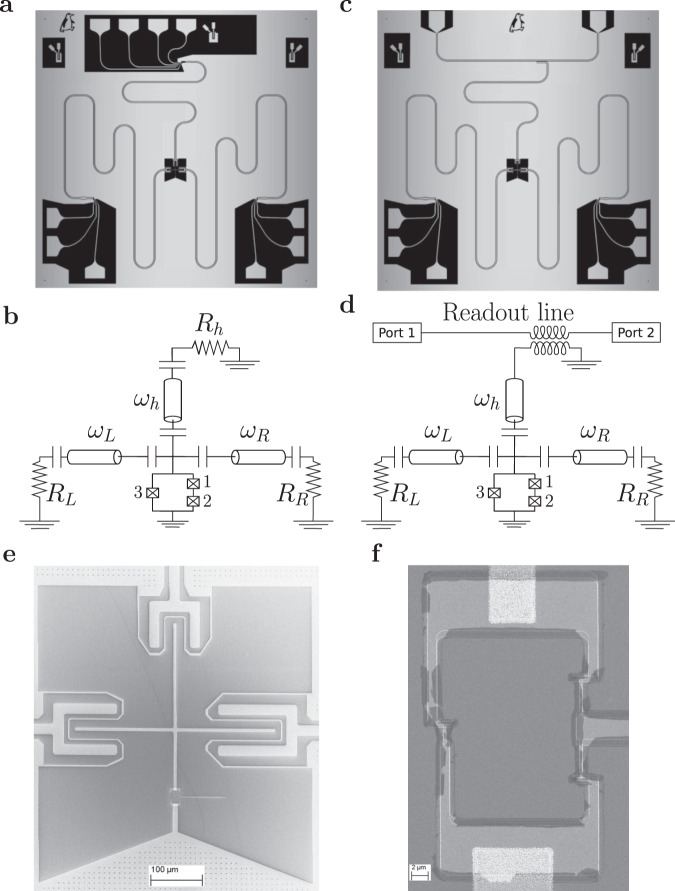
Diagrams of the samples with qubits used for the heat transport and transmission coefficient measurements. Images of the sample I used in the heat transport experiment (**a**) and of the sample II used for the microwave spectroscopy (**c**). Both samples contain a cross-shaped Xmon-type superconducting qubit, which is capacitively coupled to three superconducting coplanar waveguide resonators. Samples size 7 × 7 mm. **b** Equivalent lumped-element circuit of the sample I and (**d**) of the sample II. **e** Scanning electron micrograph of the Xmon qubit island (scale bar: 100 *μ*m) and (**f**) of the flux qubit loop with three Josephson junctions (scale bar: 2 *μ*m).

In order to fully characterize the system, we have fabricated two samples with nominally identical parameters on the same wafer, but designed for different measurement setups. The parameters of these samples may differ due to fabrication uncertainties, which we roughly estimate as 10%. The first of these devices, sample I shown in Fig. 2a, contains three resistors and was used for DC measurements of the photonic heat currents between the resistors. In the second device, sample II presented in Fig. 2c, we have replaced one of the resistors by a transmission line, which allowed us to carry out detailed spectroscopic measurements of the qubit in the microwave frequency range.

**Fig. 3:**
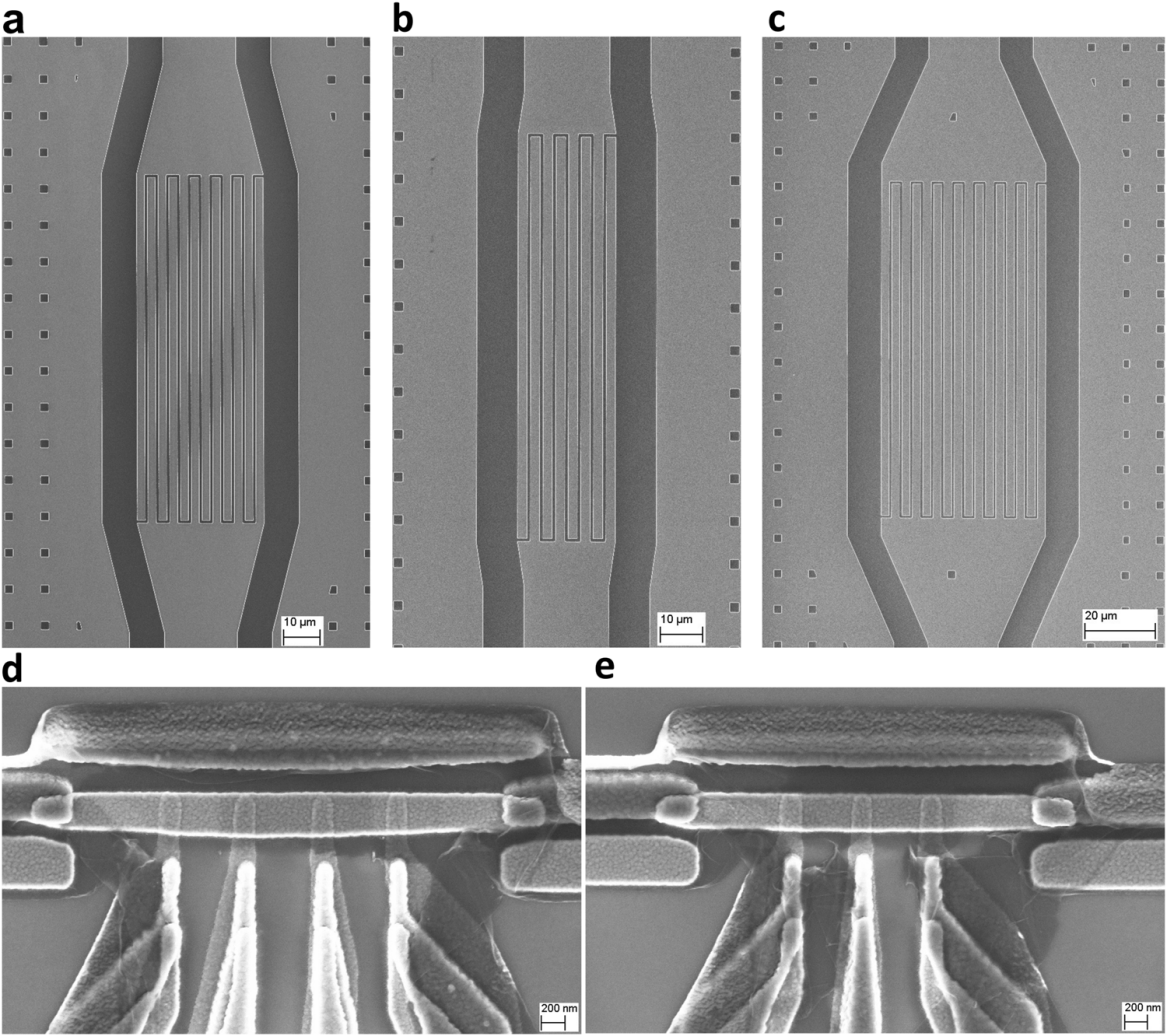
Scanning electron micrograph of capacitors and copper resistors. Scanning electron micrograph of the capacitors between resonators and right resistor ${C}_{R}^{\prime}=48fF$ (**a**) (scale bar: 10 *μ*m), hot resistor ${C}_{h}^{\prime}=30fF$ (**b**) (scale bar: 10 *μ*m), and left resistor ${C}_{L}^{\prime}=70fF$ (**c**) (scale bar: 20 *μ*m). The indicated capacitance values are obtained from COMSOL simulations. **d** Hot resistor *R*_*h*_ = 5 Ω (**d**) (scale bar: 200 *μ*m). **e** Left and right resistors have the same design and nominal values *R*_*L*_ = *R*_*R*_ = 4 Ω (**e**) (scale bar: 200 *μ*m).

Sample I contains centrally located cross-shaped superconducting island, which is capacitively coupled to three superconducting *λ*/2 coplanar waveguide (CPW) resonators with the characteristic impedance *Z*_0_ = 50 Ω as schematically shown in Fig. 2b. The bottom leg of the cross is connected to the qubit, see Fig. 2e, which is realized as a superconducting loop with three asymmetrically arranged Josephson junctions, similar to the conventional persistent-current flux qubit^25^. The SEM image of the three junction loop is presented in Fig. 2f. The top resistor, *R*_*h*_, is heated by DC bias current and serves as the heater for the whole system. Two other resistors, *R*_*L*_ and *R*_*R*_, are passively heated by the power emitted by the hot resistor and their temperatures are monitored. The resistors are realized as copper islands with the resistances *R*_*L*_ = *R*_*R*_ = 4 Ω and *R*_*h*_ = 5 Ω and the volumes ${{{{{{{{\mathcal{V}}}}}}}}}_{L}={{{{{{{{\mathcal{V}}}}}}}}}_{R}=0.036\,\mu$m^3^ and ${{{{{{{{\mathcal{V}}}}}}}}}_{h}=0.048\,\mu$m^3^. Their images are shown in Fig. 3d, e. The coupling between the qubit and the resonators is mediated by the capacitors with the designed values *C*_*L*_ = 3.81 fF, *C*_*h*_ = 4.51 fF, and *C*_*R*_ = 4.66 fF. Additional capacitors having designed values ${C}_{L}^{\prime}=70$ fF, ${C}_{h}^{\prime}=30$ fF and ${C}_{R}^{\prime}=48$ fF are inserted between the resonators and the resistors in order to keep the quality factors of the resonators sufficiently high. Their images are shown in Fig. 3a–c. The quality factors of the resonators are given as ${Q}_{i}=\pi /(2{\omega }_{i}^{2}{Z}_{0}{R}_{i}{C^{\prime} }^{2})$ for *i* = L, R or h^26^, where *ω*_*i*_ = 2*πf*_*i*_ are the resonator frequencies. With the parameters listed above and with measured resonator frequencies we estimate *Q*_*L*_ = 2450, *Q*_*R*_ = 4690 and *Q*_*h*_ = 2720. Finally, the capacitances to the ground, schematically shown in Fig. 2b, are found to be *C*_*gL*_ = 48.8 fF, *C*_*gR*_ = 54 fF, *C*_*gh*_ = 60.4 fF and *C*_*g*_ = 42 fF. All capacitance values listed above, as well as the self-capacitance of the Xmon island^27^ and the design of the SQUID are optimized by device simulation using finite element modeling COMSOL Multiphysics software packet. The designed value for the charging energy is *E*_*C*_/*h* = 0.3 GHz, and for the Josephson energy - *E*_*J*_/*h* = 4.6 GHz. The latter value corresponds to the critical current of a single junction *I*_*C*_ = 9.3 nA. The resonators are designed in such a way that the frequencies of the left and the right resonators are close to each other and to the qubit frequency, while the frequency of the hot resonator is approximately two times higher, see the Spectroscopy section for details.

As shown in Fig. 3d, e, each normal metal (N) resistor has aluminum superconducting (S) probes, separated by a thin insulating (I) layer, which we use for thermal control and readout. Four superconducting probes allow us to control and simultaneously measure the resistor temperature. We change the temperature by applying voltage bias between the two superconducting probes. For bias voltages above the superconducting energy gap the resistor is heated up, while for voltages below the gap it is cooled. The electronic temperature readout is performed by applying current bias between another pair of NIS tunnel junctions in a SINIS configuration^3^. The details of electronic thermometry are presented in the Methods section. The Andreev mirrors at the aluminum-copper boundaries on both sides of each resistor help to localize the heat, and, at the same time, they ensure good electric contact between the resistors, the ground electrode and the resonators. The dominating heat relaxation channel in the resistors is the electron-phonon coupling. Power leakage to the phonons is estimated in the usual way:

1 $${P}_{{{{{{{{\rm{el}}}}}}}}-{{{{{{{\rm{ph}}}}}}}}}={{\Sigma }}{{{{{{{\mathcal{V}}}}}}}}({T}_{{{{{{{{\rm{el}}}}}}}}}^{5}-{T}_{{{{{{{{\rm{ph}}}}}}}}}^{5}),$$

where Σ = 2 × 10^9^ Wm^−3^ K^−5^ is the electron-phonon coupling constant, ${{{{{{{\mathcal{V}}}}}}}}$ is volume of the copper resistor, *T*_el_ and *T*_ph_ are the temperatures of electrons and phonons respectively. In the steady state the power dissipated in the resistor equals to the power leaking to the phonons. Thus, measuring the electronic temperature *T*_el_ with the thermometer and knowing the substrate temperature *T*_ph_ one can easily estimate the dissipated power from Eq. (1). Alternatively, knowing Joule heating power dissipated in the hot resistor, from Eq. (1) one can estimate its electronic temperature *T*_el_.

Sample II, shown in Fig. 2c, d, and designed for the microwave spectroscopy, has nominally the same design and parameters. However, this sample does not have a hot resistor and the top resonator serves for diagnostic purposes. The latter has the same length as the top resonator of the sample I, but it is realized as *λ*/4-resonator with its upper end grounded. As a result, its frequency becomes two times lower than that in the sample I. This modification in the design brings the diagnostic resonator frequency closer to the frequencies of the two other resonators and to the transition frequency between the two lowest levels of the qubit, and makes the spectroscopy more accurate.

### Theory model

We describe the flux qubit loop containing three identical Josephson junctions with the theory model of ref. ^25^. The loop contains two superconducting islands. The first island, denoted as I1, is large and includes the cross-like aluminum electrode. It is restricted by coupling capacitors *C*_*L*_, *C*_*h*_ and *C*_*R*_ and the Josephson junctions numbered 1 and 3, as shown in Fig. 1b by red dashed line. The second island I2 is much smaller, in Fig. 1b it is indicated by the blue dashed line and sandwiched between the junctions 1 and 2. We introduce the operators *n*_*I*1_ and *n*_*I*2_ representing the number of Cooper pairs in the islands I1 and I2, and the operators *φ*_*i*_ with *i* = 1, 2 or 3, corresponding to the Josephson phase differences across the *i*th junction. The Hamiltonian of the flux qubit is^25^

2 $$H =4\mathop{\sum }_{k,l}{n}_{k}{n}_{l}{({E}_{C})}_{kl}\\ -{E}_{J}\left[\cos {\varphi }_{2}+\cos {\varphi }_{3}+\cos ({\varphi }_{{{{{{{{\rm{ext}}}}}}}}}+{\varphi }_{2}-{\varphi }_{3})-3\right],$$

where *φ*_ext_ = 2*π*Φ/Φ_0_, Φ is the normalized magnetic flux threading the loop, Φ_0_ = *h*/2*e* is the magnetic flux quantum, *E*_*J*_ = *ℏI*_*C*_/2*e* is the Josephson energy of a single junction having the critical current *I*_*C*_, ${({E}_{C})}_{kl}={e}^{2}{({C}^{-1})}_{kl}/2$, and ${({C}^{-1})}_{kl}$ are the elements of the inverse of the 2 × 2 capacitance matrix

3 $$C=\left[\begin{array}{cc}{C}_{I1}&-C\\ -C&{C}_{I2}\end{array}\right].$$

Here *C*_*I*1_ = *C*_*L*_ + *C*_*h*_ + *C*_*R*_ + *C*_*g*_ + 2*C* and *C*_*I*2_ = 2*C* are the total capacitances of the islands 1 and 2, respectively. We diagonalize the Hamiltonian (Eq. 2) numerically in the basis of two dimensional plane waves having the form $\exp (-i{n}_{I1}{\varphi }_{3}-i{n}_{I2}{\varphi }_{2})/2\pi$. Since *E*_*J*_ ≫ *E*_*C*_ = *e*^2^/2*C*_*I*1_ for all values of Φ except the narrow region close to Φ_0_/2, we neglect weak dependence of the eigen-energies *E*_*n*_ of the Hamiltonian (Eq. 2) on the gate charges induced by, for example, charged impurities.

In order to fit the experimental data on the heat power transmitted between the resistors we use the Landauer formula for the total power *P*_*i*_ carried by photons and dissipated in the resistor with the number *i*,

4 $${P}_{i}^{{{{{{{{\rm{ph}}}}}}}}}=\mathop{\sum}\limits_{j\ne i}{P}_{ij}^{{{{{{{{\rm{ph}}}}}}}}},$$

5 $${P}_{ij}^{{{{{{{{\rm{ph}}}}}}}}}=\int\nolimits_{0}^{\infty }\frac{d\omega }{2\pi }{\tau }_{ij}(\omega )\left[\frac{\hslash \omega }{{e}^{\frac{\hslash \omega }{{k}_{B}{T}_{j}}}-1}-\frac{\hslash \omega }{{e}^{\frac{\hslash \omega }{{k}_{B}{T}_{i}}}-1}\right].$$

Here the indexes *i* and *j* enumerate the resistors, i.e. they can take the values *L*, *R* or *h*, and *T*_*i*_ are the resistor temperatures. Here we have also introduced the heat currents ${P}_{ij}^{{{{{{{{\rm{ph}}}}}}}}}$ flowing from the resistor *j* to the resistor *i*. In order to derive the expression for the photon transmission probabilities *τ*_*ij*_(*ω*), we linearize Josephson dynamics and replace all three junctions in the loop by the identical inductors with the inductance *L* = *ℏ*/2*eI*_*C*_. Solving the corresponding Kirchhoff equations with thermal Nyquist noise sources connected in parallel with the resistors, as outlined in ref. ^28^ for example, we find

6 $${\tau }_{ij}(\omega )=\frac{{{{{{{{\rm{Re}}}}}}}}\left[\frac{1}{{Z}_{i}(\omega )}\right]{{{{{{{\rm{Re}}}}}}}}\left[\frac{1}{{Z}_{j}(\omega )}\right]}{| \frac{1}{{Z}_{J}(\omega )}+\frac{1}{{Z}_{L}(\omega )}+\frac{1}{{Z}_{R}(\omega )}+\frac{1}{{Z}_{h}(\omega )}{| }^{2}}.$$

Here we have introduced the impedances of the three segments of the electric circuit depicted in Fig. 1b, which contain individual resonators, resistors and coupling capacitors. They are defined as

7 $${Z}_{j}(\omega )=\frac{1}{-i\omega {C}_{j}}+\frac{1}{-i\omega {C}_{gj}+{Z}_{rj}^{-1}(\omega )},$$

where *Z*_*rj*_(*ω*) have the form

8 $${Z}_{rj}(\omega )={Z}_{0}\frac{\left({R}_{j}+\frac{1}{-i\omega {C}_{j}^{\prime}}\right)\cos \omega {t}_{j}-i{Z}_{0}\sin \omega {t}_{j}}{-i{Z}_{0}\cos \omega {t}_{j}-i\left({R}_{j}+\frac{1}{-i\omega {C}_{j}^{\prime}}\right)\sin \omega {t}_{j}}.$$

Here *t*_*j*_ is the flight time of a photon between the two ends of a given resonator which is proportional to its length. The impedance of the three junction loop for the flux values ∣Φ∣ ≤ Φ_0_/2 takes the form

9 $$\frac{1}{{Z}_{J}(\omega )}=-i\omega \left({C}_{g}+\frac{3}{2}C\right)+\frac{3e{I}_{C}}{-i\hslash \omega }\cos \left(\frac{2\pi }{3}\frac{{{\Phi }}}{{{{\Phi }}}_{0}}\right),$$

and it should be periodically extended with the period Φ_0_ for ∣Φ∣ > Φ_0_/2. Transmission probabilities (Eq. 6) exhibit multiple peaks centered at frequencies corresponding to the modes of the resonators and one additional narrow peak at the resonance frequency of the three junction loop

10 $${\omega }_{0}({{\Phi }})=\sqrt{\frac{3e{I}_{C}}{\hslash {C}_{{{\Sigma }}}}\cos \left(\frac{2\pi }{3}\frac{{{\Phi }}}{{{{\Phi }}}_{0}}\right)},\ \ | {{\Phi }}| \, < \, \frac{{{{\Phi }}}_{0}}{2}.$$

Here *C*_Σ_ = *C*_*g*_ + 3*C*/2 + *C*_*L*_ + *C*_*R*_ + *C*_*h*_ is effective capacitance of the qubit, which is similar, but slightly different from *C*_*I*1_. The frequency *ω*_0_ is close to the exact transition frequency between the two lowest levels of the non-linear qubit 2*πf*_01_ = (*E*_1_ − *E*_0_)/*ℏ*, but deviates from it in the vicinity of the flux point Φ = Φ_0_/2, where qubit anharmonicity becomes significant.

As shown in ref. ^28^, the Landauer formula ((Eq. 4), (Eq. 5)) can be derived from the Kirchhoff’s equations relating the Fourier components *I*_*i*,*ω*_ of the four fluctuating in time currents *I*_*i*_(*t*), which flow from the central cross shaped island of the device in the left (*i* = *L*), the hot (*i* = *h*), the right (*i* = *R*) resonators and in the qubit (*i* = *q*), with the island potential *V*_*isl*_,

11 $${I}_{i,\omega }=\frac{{V}_{isl,\omega }}{{Z}_{i}(\omega )}+{\xi }_{i\omega }.$$

Here *ξ*_*i*,*ω*_ are the Fourier components of the noise currents with the spectral densities determined by the fluctuation-dissipation theorem,

12 $$\langle | {\xi }_{i,\omega }{| }^{2}\rangle ={{{{{{{\rm{Re}}}}}}}}\left[\frac{1}{{Z}_{i}(\omega )}\right]\hslash \omega \coth \frac{\hslash \omega }{2{k}_{B}{T}_{i}}.$$

These noises are generated by the resistors *R*_*i*_ and acquire the spectrum (Eq. 12) close to the island due to the filtering effect of the resonators. The qubit does not generate any noise, which means *ξ*_*q*,*ω*_ = 0. Equation (11) should be supplemented by the current conservation condition

13 $$\mathop{\sum}\limits_{j}{I}_{j,\omega }=0.$$

The power dissipated in the resistor *R*_*i*_ is expressed as

14 $${P}_{i}=\langle {I}_{i}(t){V}_{isl}(t)\rangle =\int \frac{d\omega }{2\pi }\langle {I}_{i,\omega }{V}_{isl,\omega }^{* }\rangle .$$

Solving Eqs. (11), (13) we express the currents *I*_*j*,*ω*_ and the potential *V*_*isl*,*ω*_ via the three noises *ξ*_*L*_, *ξ*_*h*_ and *ξ*_*R*_. Substituting the result in Eq. (14) and taking the averages with the aid of Eq. (12), we arrive at Eqs. (4), (5).

As we mentioned, Eqs. (5), (6) rely on replacing the non-linear Josephson junctions by linear inductors. This approximation is formally valid in the two limits: at low temperatures *k*_*B*_*T*_*i*_ ≪ *U*_*b*_ and at high temperatures *k*_*B*_*T*_*i*_ ≫ *U*_*b*_, where *U*_*b*_ is the height of the potential barrier in the potential (Eq. 2) varying from *U*_*b*_ = 4*E*_*J*_ at Φ = 0 to *U*_*b*_ = *E*_*J*_/2 at Φ = 0.5Φ_0_. In the former case, Josephson phases fluctuate close to the bottom of the potential well where one can use harmonic approximation; in the latter case one can put *E*_*J*_ = 0, which again makes the system linear and the Landauer formula (Eq. 5), (Eq. 6) valid. In the intermediate regime *k*_*B*_*T*_*i*_ ~ *U*_*b*_ the non-linearity of the junctions is important, but even in this case Eqs. (5), (6) reasonably well describe the heat transport^29^.

### Microwave spectroscopy

**Fig. 4:**
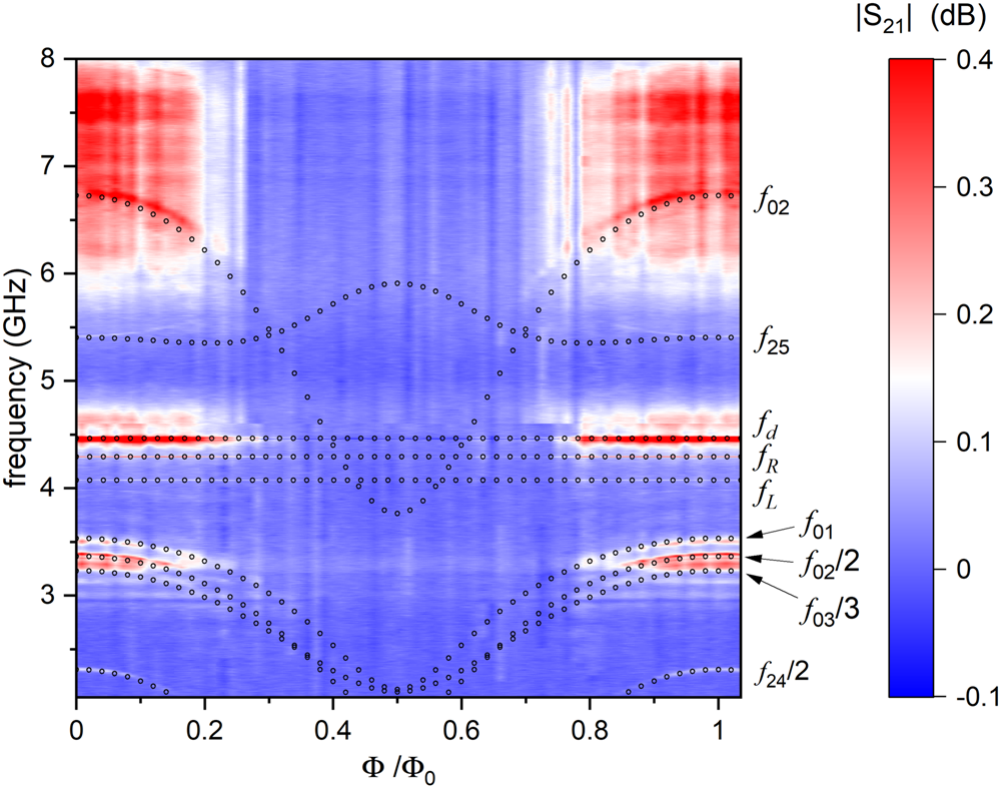
Results of the two-tone spectroscopy with simulations to match the qubit transitions. Experimentally measured transmission coefficient ∣*S*_21_∣ as a function of the normalized magnetic flux Φ/Φ_0_ and of the frequency *f*. Cross symbols indicate the modes of the resonators and the dotted lines are the theory predictions for the single and two photon interlevel transitions.

In order to obtain accurate information about the device parameters, we performed microwave spectroscopy on the Sample II. In Fig. 4 we show the results of the two-tone spectroscopy and plot the absolute value of the transmission coefficient ∣*S*_21_∣ between the ports 2 and 1, shown in Fig. 2d, as a function of the magnetic flux Φ and the frequency of the probe signal. The spectroscopy reveals a series of lines. In particular, we observe single photon transitions at frequencies *f*_01_, *f*_12_, *f*_02_ and *f*_25_, where *f*_*ij*_ = (*E*_*j*_ − *E*_*i*_)/*h* is the transition frequency between the energy levels of the qubit *E*_*i*_ and *E*_*j*_, and several two-photon transitions corresponding to the frequencies *f*_02_/2, *f*_24_/2.

Next, we compare the experimental results with the theory model based on the Hamiltonian (Eq. 2). Adjusting the model parameters, we have managed to fit the positions of the experimental spectral lines with high accuracy. In this way, we have obtained the total capacitance of the Xmon island *C*_*I*1_ = 70.26 fF and the junction capacitances *C* = *C*_*I*2_/2 = 4.13 fF. From the same fit we have also obtained the values of the Josephson energy of a single junction, *E*_*J*_/*h* = 4.86 GHz and the charging energy of the island I1, *E*_*C*_/*h* = 276 MHz. The errors in the fit is estimated as 3% for *E*_*J*_ and 4% for capacitance *C*_*I*1_ − 2*C*_*J*_. All these values are close to the designed ones. In addition, microwave spectroscopy provides accurate values of the resonator frequencies, *f*_*L*_ = 4.074 GHz, *f*_*R*_ = 4.292 GHz, and the frequency of the diagnostic *λ*/4-resonator *f*_*d*_ = 4.464 GHz with its quality factor *Q*_*d*_ = 935. The resonator lines shown in Fig. 4 have small dispersive shifts induced by the coupling to the qubit, however, they are not visible on the scale of the figure. For example, for the right resonator we obtained the dispersive shift *χ*_*R*_ = 1.8 MHz. Importantly, the qubit frequency *f*_01_(Φ) does not cross the frequencies of the resonators and stays below them for all values of the magnetic flux. In this regime the coupling constants between the resonators and the qubit can be estimated from the dispersive shifts as ${g}_{j}=\sqrt{({f}_{j}-{f}_{01}^{\max }){\chi }_{j}}$, where *j* = *L*, *R*, *d* and ${f}_{01}^{\max }=3.6$ GHz is the maximum 0 ↔ 1 transition frequency. In this way we obtain *g*_*R*_ = 35 MHz. In the next section we use these parameters to describe the heat transport in the Sample I, which has been fabricated in the same way. However, sample to sample scattering of the capacitances and the junction resistances during the fabrication can reach 10%, therefore we slightly adjust the capacitance *C*_*gR*_ to achieve better fit.

**Fig. 5:**
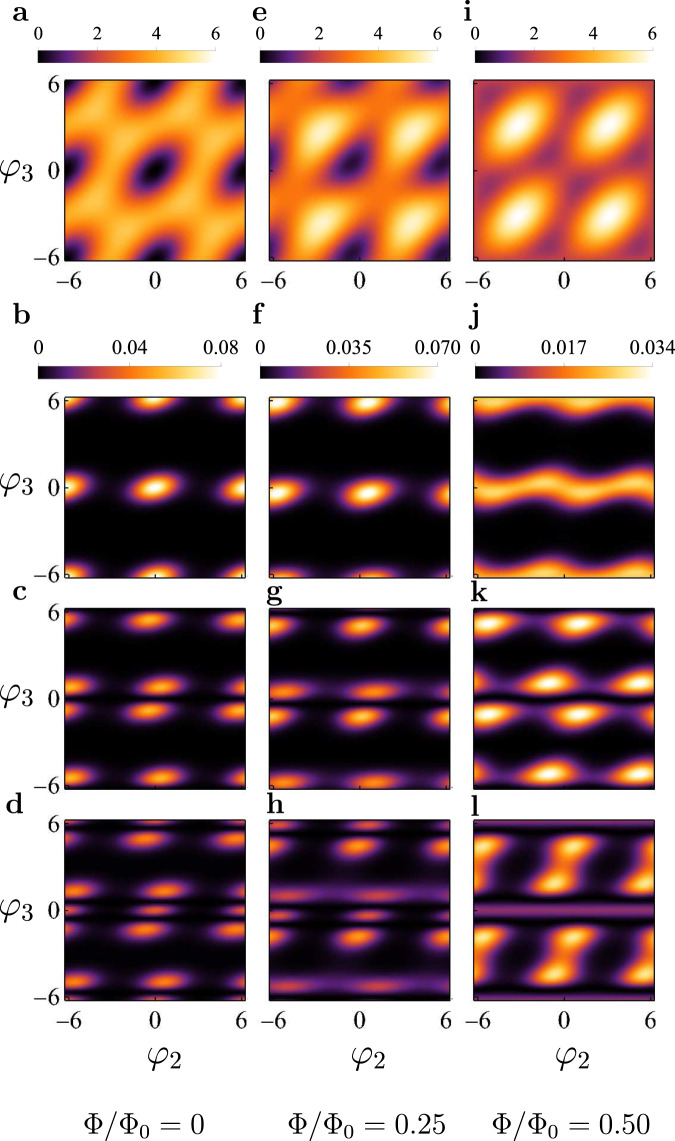
Potential energy and the square of the wave functions corresponding to the first three energy levels. Two dimensional potential of the flux qubit for three different values of the magnetic flux: Φ = 0 (**a**), Φ = 0.25Φ_0_ (**e**) and Φ = 0.5Φ_0_ (**i**). The squared wave functions of the ground state with *n* = 0 are shown in **b**, **f**, **j**; of the first excited level with *n* = 1—in **c**, **g**, **k**; and the second excited level with *n* = 2—in **d**, **h**, **l**.

Having determined the system parameters from the fits, in Fig. 5 we plot the two-dimensional potential of the three junction loop, defined in Eq. (2), for three values of the magnetic flux, Φ/Φ_0_ = 0, 0.25 and 0.5. In the same figure we also plot the squared absolute values of the wavefunctions of the three lowest energy levels. We observe that at flux values Φ = 0 and Φ = 0.25Φ_0_ the wavefunction of the *n*th level shows *n* nodes in the *φ*_2_ direction, as expected for a one-dimensional potential. Thus, in this case the two-dimensional wave function can be approximately factorized into the product Ψ_*n*_(*φ*_2_, *φ*_3_) ≈ *ψ*_*n*_(*φ*_2_)*ψ*_0_(*φ*_3_), where *ψ*_0_(*φ*_3_) is the ground state wave function in *φ*_3_ direction. Since such factorization is exact for a harmonic potential well, we conclude that at these flux values the linearized model of the qubit, on which Eqs. (5), (6) are based, should work reasonably well. However, at Φ = 0.5Φ_0_ this approximation is no longer accurate because the potential well becomes shallow and hence anharmonic.

### Photonic heat transport

**Fig. 6:**
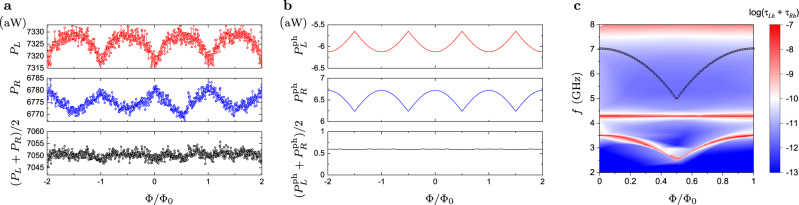
Modulation of heat powers with heat transport and photon transmission modeling. **a** Experimentally observed flux dependent heat powers dissipated in the left resistor (*P*_*L*_, red dots), in the right resistor (*P*_*R*_, blue dots) and the average value (*P*_*L*_ + *P*_*R*_)/2 (black dots). Heating voltage *V*_*h*_ = 0.9 mV has been applied to the hot resistor, which resulted in the resistor temperatures *T*_*h*_ = 393 mK, *T*_*L*_ = 168 mK and *T*_*R*_ = 164 mK. The temperature of the mixing chamber was *T*_MXC_ = 127 mK. **b** Theory prediction, based on Eqs. (4), (5), for the photonic heat powers dissipated in the left (${P}_{L}^{{{{{{{{\rm{ph}}}}}}}}}$ red line) and the right (${P}_{R}^{{{{{{{{\rm{ph}}}}}}}}}$ blue line) resistors. Black line in the bottom panel is the average value $({P}_{L}^{{{{{{{{\rm{ph}}}}}}}}}+{P}_{R}^{{{{{{{{\rm{ph}}}}}}}}})/2$. We assumed the following resistor temperatures: *T*_*h*_ = 393 mK, *T*_*L*_ = 176 mK and *T*_*R*_ = 156 mK. **c** The sum of the photon transmission probabilities *τ*_*Lh*_(*ω*, Φ) + *τ*_*Rh*_(*ω*, Φ), given by Eq. (6), assuming the same fit parameters as in the (**b**). Black circles indicate the double frequency of the three junction loop, 2*ω*_0_(Φ)/(2*π*), where *ω*_0_(Φ) is defined in Eq. (10).

**Fig. 7:**
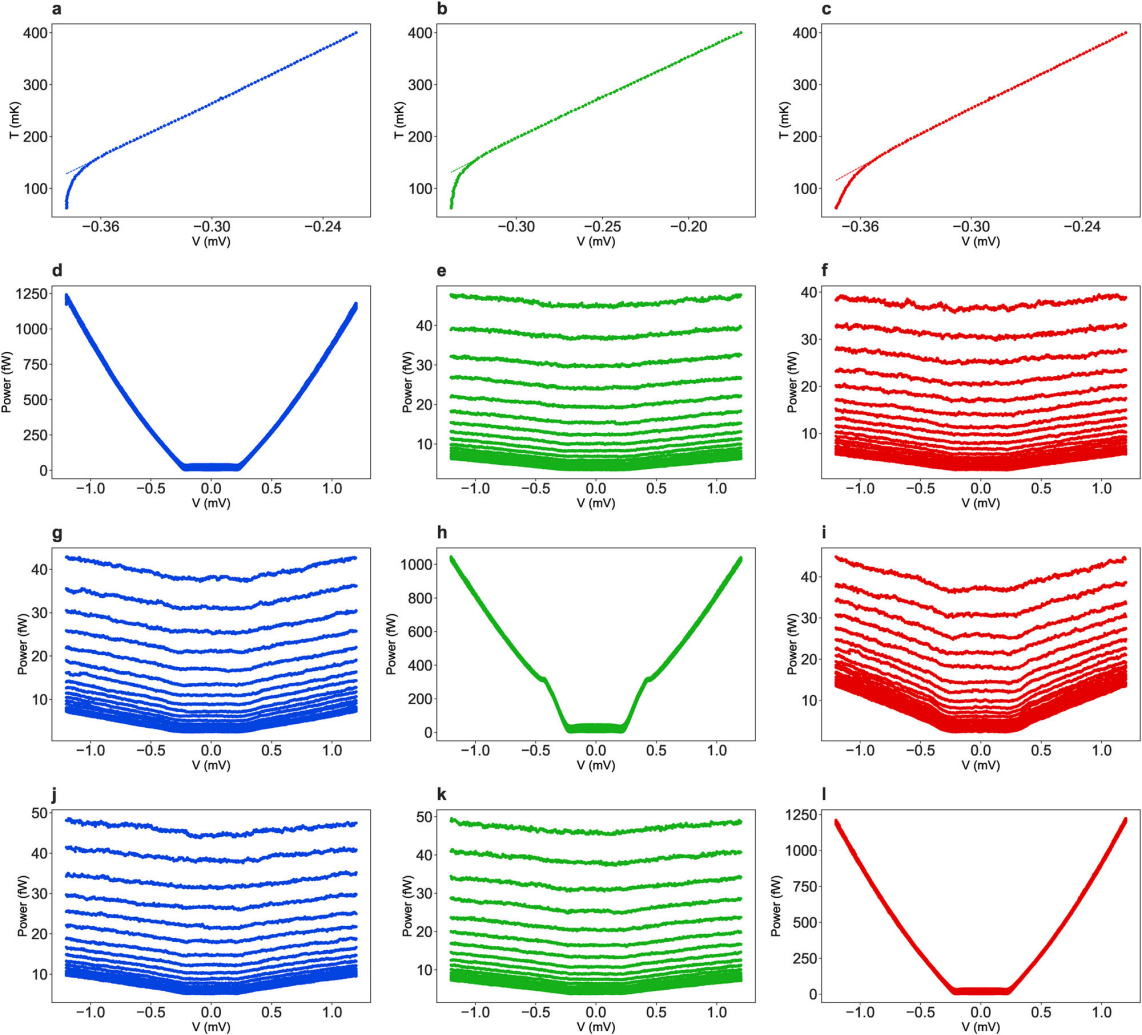
Experimentally measured electron temperature of the SINIS thermometers. Calibration of the SINIS thermometer connected to the left resistor *R*_*L*_ indicated by blue (**a**), to the right resistor *R*_*R*_—green (**b**) and to the hot resistor *R*_*h*_—red (**c**). The dashed lines indicate the polynomial data fitting. In all these plots the horizontal axis shows the voltage drop across the SINIS structure at fixed bias current and the vertical axis—the temperature of the mixing chamber. Figures **d**, **h**, **l** show the dependence of the power dissipated in, respectively, left, right and hot resistors versus heating voltage applied to the same resistor. Figures **g**, **j** show the non-local response in the system, where the power dissipated in the left resistor is plotted versus the heating voltages applied to the right (**g**) and to the hot (**j**) resistors. We also plot the non-local response of the right resistor on the bias voltages applied to left (**e**) and to the hot ((**k**) resistors, and of the hot resistor on the bias applied to left (**f**) and right (**i**) resistors.

We performed heat transport measurements in Sample I, which has nominally identical parameters with Sample II. The hot resistor was heated up by the DC voltage *V*_*h*_, and the temperatures of the left and the right resistors were monitored with SINIS thermometers, as it was explained above. We will focus on the data taken at the heating voltage *V*_*h*_ = 0.9 mV and plotted in Fig. 6a as functions of the magnetic flux Φ. First, we have estimated the power dissipated in the hot resistor as *P*_*h*_ = *I*_*h*_*V*_*h*_/2 (see the calibration curve in Fig. 7l measured on a different device, which has parameters identical to Sample I), and further using Eq. (1) we have found the temperature of the hot resistor, *T*_*h*_ = 393 mK. This temperature is rather high and, therefore, cannot be accurately measured by the thermometer. The temperatures of the left and of the right resistors, which were lower, have been directly measured by SINIS thermometers and have been found to be *T*_*L*_ = 168 mK and *T*_*R*_ = 164 mK. Varying the magnetic flux applied to the SQUID loop, we have observed small oscillations of these temperatures with the amplitudes *δT*_*L*,*R*_ ~ 0.01 mK. The measured temperatures have been converted to powers dissipated in the left (*P*_*L*_) and in the right (*P*_*R*_) resistors using Eq. (1). *P*_*L*_(Φ) and *P*_*R*_(Φ) oscillate with the period Φ_0_ following the qubit frequency *f*_01_(Φ) shown in Fig. 4. These oscillations have opposite signs for the two resistors, i.e when *P*_*L*_ increases *P*_*R*_ goes down and vice versa. The oscillation amplitudes are similar for both resistors, that is why the average value (*P*_*L*_ + *P*_*R*_)/2, shown by black dots, is almost constant. This interesting observation suggests that the flux dependent contributions to *P*_*L*_ and *P*_*R*_ originate from the heat current between these two resistors *P*_*LR*_. Similar behavior of the dissipated powers *P*_*L*_, *P*_*R*_ has been observed for other values of the heating voltage *V*_*h*_.

In order to understand the flux dependence of the powers *P*_*L*_ and *P*_*R*_ better, we have numerically evaluated the photonic heat currents ((Eq. 4), (Eq. 5)) for a circuit depicted in Fig. 2b. Theoretical results are very sensitive to the parameter values. For example, the variation of the capacitances *C*_*L*_ and *C*_*R*_ within 10%, which is the estimated fabrication error, may change the heat fluxes by the factor 10^3^–10^4^. The origin of such sensitivity is simple—any mismatch between the narrow spectral lines of the resonators and of the qubit strongly reduces the heat flow. For this reason, we do not aim at the perfect fit, our goal is to show that with the nominal parameters of the experiment the theoretical model produces qualitatively similar results. Thus, for this simulation we have used the values of the resistances and capacitances given in sections Experiment and Spectroscopy. We have made only few adjustments of the parameters in order to increase the oscillation amplitudes of the heat powers *P*_*L*_, *P*_*R*_. Namely, we have chosen slightly larger value for the ground capacitance *C*_*gR*_ = 70.5 fF and thus brought the right resonator in resonance with the left one, so that *f*_*L*_ = *f*_*R*_ = 4.292 GHz. The frequency of the hot resonator was taken to be *f*_*h*_ = 8.237 GHz, i.e. it is two times higher than the frequency of the diagnostic resonator of the Sample II. We have also increased the temperature difference between the left and the right resistors by choosing *T*_*L*_ = 176 mK and *T*_*R*_ = 156 mK. Such increase in *T*_*L*_ − *T*_*R*_ is within the experimental uncertainty. Finally, we have reduced the Josephson energy to *E*_*J*_/*h* = 3.63 GHz to achieve better agreement between spectroscopy plots of Figs. 4 (experiment) and 6c (theory). Photonic heat powers ${P}_{L}^{{{{{{{{\rm{ph}}}}}}}}}({{\Phi }})$ and ${P}_{R}^{{{{{{{{\rm{ph}}}}}}}}}({{\Phi }})$, obtained in this way, are plotted in Fig. 6b. They indeed behave similarly to the experimental ones, namely, they oscillate in opposite directions and the average power $({P}_{L}^{{{{{{{{\rm{ph}}}}}}}}}+{P}_{R}^{{{{{{{{\rm{ph}}}}}}}}})/2$ is almost independent of the flux. Theory modeling clarifies the origin of this effect. Indeed, according to Eq. (4) the photonic heat powers dissipated in the resistors are expressed as ${P}_{L}^{{{{{{{{\rm{ph}}}}}}}}}={P}_{Lh}^{{{{{{{{\rm{ph}}}}}}}}}+{P}_{LR}^{{{{{{{{\rm{ph}}}}}}}}}$, ${P}_{R}^{{{{{{{{\rm{ph}}}}}}}}}={P}_{Rh}^{{{{{{{{\rm{ph}}}}}}}}}-{P}_{LR}^{{{{{{{{\rm{ph}}}}}}}}}$. Numerically we find that the currents flowing from the hot resistor to the left and the right ones, ${P}_{Lh}^{{{{{{{{\rm{ph}}}}}}}}}$ and ${P}_{Rh}^{{{{{{{{\rm{ph}}}}}}}}}$, weakly depend on magnetic flux because of the strong detuning between the qubit and the hot resonator. Thus, the flux dependence of the powers ${P}_{L}^{{{{{{{{\rm{ph}}}}}}}}}$ and ${P}_{R}^{{{{{{{{\rm{ph}}}}}}}}}$ predominantly comes from the photonic heat current flowing from the right to the left resistor ${P}_{LR}^{{{{{{{{\rm{ph}}}}}}}}}({{\Phi }})$, which contributes to them with opposite signs due to energy conservation. Comparing Fig. 6a and b, we notice the similarity in the shape of the experimental and the theoretical power-flux dependences for the right resistor. However, the theoretical curve for the left resistor is inverted and shifted by one half of the flux quantum relative to the experimental one. By tuning the system parameters further one can, in principle, reproduce the right shape of the ${P}_{L}^{{{{{{{{\rm{ph}}}}}}}}}({{\Phi }})$ dependence.

In Fig. 6c we plot the sum of the two transmission probabilities *τ*_*Lh*_(*ω*, *ϕ*) + *τ*_*Rh*_(*ω*, Φ) (Eq. 6) evaluated with the same fit parameters as in the theory plots of Fig. 6b. We note close resemblance of this plot with Fig. 4, in which the measured value of the transmission coefficient ∣*S*_21_(*ω*, Φ)∣ is presented. This similarity is expected because the two values are approximately related as ∣*S*_21_∣^2^ ≈ 1 − *α*(*τ*_*Lh*_ + *τ*_*Rh*_), where the constant *α* depends on the system parameters. It also demonstrates that our model rather accurately describes the system even though it neglects the anharmonicity of the qubit. In addition, Fig. 6c helps to clarify the origin of the unusual non-sinusoidal shape of the power oscillations in Fig. 6a, b. Indeed, since the qubit and the resonator frequencies do not cross each other and the contribution of the qubit spectral lines to the heat transport is quite weak, one can rather accurately expand the heat current ${P}_{LR}^{{{{{{{{\rm{ph}}}}}}}}}$ in powers of the resonance frequency *ω*_0_(Φ), ${P}_{LR}({{\Phi }})\approx {P}_{LR}^{(0)}+A{\omega }_{0}^{2}({{\Phi }})$, where *A* is a pre-factor. The last term in this expression $\propto {\omega }_{0}^{2}({{\Phi }})$ produces the cusps visible in *P*_*L*,*R*_(Φ) dependencies both in Fig. 6a, b. They occur at Φ = Φ_0_/2 where the dependence *ω*_0_(Φ) also has a cusp, see the lowest yellow line in Fig. 6c. The cusps become a bit rounded if one proceeds more accurately and replaces the frequency *ω*_0_(Φ)/2*π* by the qubit transition frequency *f*_01_(Φ) in the expansion. At the flux point Φ = 0.5Φ_0_ the harmonic approximation becomes inaccurate. Indeed, at this point the qubit frequency *ω*_*J*_/2*π* is a bit higher than the frequency *f*_01_ computed with full quantum approach, cf. (Figs. 4 and 6c).

The absolute values of experimentally measured (Fig. 6a) powers exceed the numerically estimated ones (Fig. 6b) by a factor ~10^3^. Thus, most of the heat power between the resistors is transmitted by the substrate phonons or by other mechanisms not included in our model. It is typical for this type of experiments, see e.g. refs. ^12,13^. Indeed, one has to heat the system strongly to make the small photonic heat flux measurable, but other contributions to the heat flux grow even stronger with rising temperature. The theoretical power modulation amplitudes also differ the experimental ones: we find *δP*_*L*_ ≈ 13 aW and *δP*_*R*_ ≈ 10 aW in the experiment, and $\delta {P}_{L}^{{{{{{{{\rm{ph}}}}}}}}}=\delta {P}_{R}^{{{{{{{{\rm{ph}}}}}}}}}\approx 0.54$ aW in the simulation. In theory one can increase the power modulation by, for example, increasing the qubit frequency so that it crosses the frequencies of the resonators. In general, we have noticed that the model systematically underestimates the modulation amplitude of the powers *P*_*L*_ and *P*_*R*_. Further research is required in order to resolve this problem.

## Discussion

We have studied the heat transport by photons in a three terminal system containing a flux qubit realized as superconducting loop with three identical Josephson junctions. We have combined the DC heat transport measurements with the microwave spectroscopy performed on a separate sample with nominally identical parameters. In this way, we have verified that the flux qubit operates as a quantum system with its level spacing modulated by magnetic flux, and related this effect to the modulation of the heat power in DC measurements. Employing the standard theory models, we have described both the changes in the qubit spectrum and the modulation of the heat flux by magnetic field with the same set of parameters. Our experiment is an important step towards practical realization of on-chip quantum heat transistors, thermal amplifiers and heat pumped masers.

## Methods

Fabrication. The devices are fabricated using three steps of electron-beam lithography (EBL) to create a mask on a silicon substrate. First EBL mask to prepare the niobium pattern and the groundplane of the device: feedline, resonators, cross-shape island and superconducting probes for thermometry are formed by reactive ion etching a 200 nm niobium metal film deposited by DC magnetron sputtering onto the high-resistivity silicon wafer. A 20-nm-thick aluminum film has been grown by atomic layer deposition on the wafer prior to Nb sputtering. The characteristic impedance of the coplanar waveguides used for the feedline and resonators is 50 Ω. Second, the SQUID with three Josephson junctions is realized with shadow-mask EBL, formed using two layers of poly(methylmetalcrylate-methacrylic) acid P(MMA-MAA) resist spun for 60 s at 4000 rpm followed by one layer of polymethyl-metacrylate (PMMA) spun for 60 s at 4000 rpm, all resist layers are immediately baked at 160 C, and followed by thin film physical vapor deposition in an electron-beam evaporator with an intermediate oxidation, using the Dolan bridge technique. The evaporation was preceded by argon ion plasma milling to facilitate the clean contact between aluminum and niobium. Third, the normal metal resistors and the normal-insulator-superconductor (NIS) tunnel junction elements for thermometry are also patterned by EBL, using the same resist, and then similarly deposited onto the wafer in three steps: Al with an in situ oxidation to make NIS junctions, Cu for reservoirs and Al. The fabrication is completed by spin-coating a protective layer of photoresist (AZ5214E) and dicing by diamond-embedded resin blade to 7 × 7 mm size samples. The resist was then removed using acetone. The resistance of the SIS ${{{{{{{\mathcal{R}}}}}}}}=28\pm 1\,{{{{{{{\rm{k}}}}}}}}{{\Omega }}$ and NIS tunnel junctions ${{{{{{{\mathcal{R}}}}}}}}=12\pm 1\,{{{{{{{\rm{k}}}}}}}}{{\Omega }}$ was measured on the test structures of the same dimensions on the same chip as shown in Fig. 2a. Both devices for heat transport measurements (Fig. 2a) and RF characterization (Fig. 2b) were fabricated simultaneously on the same wafer.

RF spectroscopy. The sample for RF spectroscopy was cooled in a BlueFors dilution refrigerator with a base temperature of 10 mK. In this design, a 7.4 GHz spectroscopy resonator connects the top terminal of the cross-shaped island to a feedline for spectroscopy characterization via transmission microwave readout. The spectroscopy measurements were performed using a vector network analyzer (VNA) at room temperature with the signal reaching the sample via an RF line and attenuated at different temperatures inside the cryostat. This scheme is presented in Fig. 2e. The output signal is then passed through two circulators at base temperature to a low noise HEMT amplifier mounted at 4 K, providing 46 dB gain, followed by an additional 28 dB amplifier outside of the cryostat. Characterization started by measuring the transmission *S*_21_ through the feedline to identify the *λ*/4 diagnostic resonator. To characterize the qubit and qubit-resonator couplings we perform two-tone spectroscopy by first applying a microwave low-power probe tone, followed by a second tone, whose frequency is swept. The Josephson energy is tuned by applying the magnetic flux with an external coil.

Thermal conductance measurements were performed in a custom-made plastic dilution refrigerator with a mixing chamber (MXC) temperature varied in the range 90–400 mK. The device is wire-bonded in a custom-made brass stage enclosed by two brass Faraday shields and fixed to the MXC with a proper thermalization. The readout scheme consists of thermocoax-filtered cryogenic lines with effective signal bandwidth of 0–10 kHz, for low-impedance loads. At room temperature, the voltage signal is amplified by a low noise amplifier Femto DLVPA-100-F-D. Heating of the thermal reservoir is realized by DC/AC signals, which were applied by programmable function generators and read on the control thermal reservoirs by multimeter/lock-in amplifier, synchronised to the square-wave modulation *f* ~ 77 Hz of the heated voltage bias. Thermometry was performed in SINIS configuration^3^, calibration of thermometers, presented in Fig. 7a–c for left *R*_*L*_, right *R*_*R*_ and hot *R*_*h*_ resistors, was done by monitoring the voltage while applying a current bias between the superconducting probes and varying the MXC temperature up to 400 mK. The device is well thermalized to the MXC, therefore we can assume that the phonon temperature is in equilibrium with the MXC temperature, which is monitored by a ruthenium oxide thermometer that has been calibrated against a Coulomb blockade thermometer. The energy conservation among three thermal reservoirs is verified by performing three measurements: (1) heating the left resistor *R*_*L*_ and performing local thermometry on *R*_*L*_ and remote on the right resistor *R*_*R*_ and hot resistor *R*_*h*_ (Fig. 7d–f); (2) heating and local thermometry on the right resistor *R*_*R*_ and remote thermometry on the left resistor *R*_*L*_ and hot resistor *R*_*h*_ (Fig. 7g–i); (3) heating and local thermometry on the hot resistor *R*_*h*_ and remote thermometry on the left resistor *R*_*L*_ and right resistor *R*_*R*_ (Fig. 7j–l). In Fig. 6 the magnetic flux is tuned by a superconducting solenoid encompassing the entire sample stage assembly, inside of a high-permeability magnetic shield, which is mounted inside the refrigerator at 4 K.

## Data Availability

The data that support the plots within this article are available from the corresponding author upon reasonable request.

## Code availability

The codes that support the findings of this study are available from the corresponding author upon reasonable request.
